# A UHPLC-Orbitrap-MS Metabolomics Strategy Reveals Glycerophospholipid Metabolic Remodeling Is Associated with the Anti-Arthritic Effect of Glycyrrhiza Protein–Paeoniflorin Nanoparticles via PI3K/AKT/NLRP3 Axis

**DOI:** 10.3390/molecules31030554

**Published:** 2026-02-05

**Authors:** Na Zhang, Xiaoyun Yang, Cui Li, Miaoxin Huo, Yuan Gao, Dong Bai, Yuqin Yang

**Affiliations:** Institute of Basic Theory of Traditional Chinese Medicine, China Academy of Chinese Medical Sciences, Beijing 100700, China; bucmzn@163.com (N.Z.);

**Keywords:** rheumatoid arthritis, glycyrrhiza protein–paeoniflorin nanoparticles, UHPLC-Orbitrap-MS metabolomics, glycerophospholipid metabolism, PI3K/AKT/mTOR pathway

## Abstract

Rheumatoid arthritis involves chronic synovitis and immune-metabolic dysregulation, highlighting a need for multi-target therapies that jointly modulate metabolism and inflammation. We developed glycyrrhiza protein–paeoniflorin self-assembled nanoparticles (GP-PF NPs) and investigated their anti-arthritic mechanism in adjuvant-induced arthritis (AIA) mice, using UHPLC-Orbitrap-MS-based metabolomics. Male C57BL/6 mice (*n* = 42) were assigned to the control, model, GP-PF NPs, paeoniflorin, glycyrrhiza protein, physical mixture, and celecoxib groups. All groups except controls received complete Freund’s adjuvant, and treatments were given intraperitoneally for 10 days. GP-PF NPs produced the greatest reduction in paw thickness versus the model (*p* < 0.0001) and outperformed all other active treatments, which was consistent with the improved histopathology. UHPLC-Orbitrap-MS detected 473 serum metabolites, and the model group showed 59 significant changes versus the control. GP-PF NPs significantly modulated 108 metabolites and yielded robust OPLS-DA separation from the model (R^2^Y = 0.98; Q^2^ = 0.742). Venn and pathway analyses identified 43 NP-specific metabolites enriched in glycerophospholipid metabolism, including glycerophosphocholine, 1-oleylglycerophosphocholine, PE (16:0/16:0), phosphocholine, and sphingosine-1-phosphate. These metabolites were selectively normalized toward control levels by GP-PF NPs. qPCR further showed that GP-PF NPs significantly reduced synovial PI3K, AKT, mTOR, NLRP3, Caspase-1, and GSDMD mRNA overexpression (all *p* < 0.001 vs. model). Correlation analysis indicated significant associations between key serum lipids and synovial genes (e.g., PI3K positively correlated with several metabolites, r = 0.71–0.82; mTOR negatively correlated with sphinganine 1-phosphate and glycerophosphocholine, r = −0.65 and −0.54). These data suggest that GP-PF NPs ameliorate AIA and are associated with the normalization of glycerophospholipid-related metabolic perturbations and reduced synovial mRNA expression of the PI3K/AKT/mTOR-NLRP3 pathway, supporting their potential as a metabolism-inflammation preclinical oriented anti-arthritic nanomedicine.

## 1. Introduction

Rheumatoid arthritis (RA) is a chronic, systemic autoimmune disorder characterized by persistent synovitis, which results from the infiltration of immune cells and the release of proinflammatory mediators, ultimately leading to progressive cartilage and bone destruction [1]. Globally, RA affects approximately 0.5% to 1% of the population, making it a leading cause of disability and reduced work capacity, with significant socioeconomic consequences [2,3]. Despite the substantial clinical improvements achieved with traditional synthetic disease-modifying antirheumatic drugs (DMARDs), such as methotrexate, and the advent of biologic agents and targeted synthetic DMARDs, a considerable proportion of patients still experience inadequate initial responses, drug resistance, or adverse drug reactions [4,5]. Consequently, the development of novel anti-inflammatory agents that can modulate multiple pathways and targets in a synergistic manner remains a critical focus for RA treatment.

Recent advances in immunometabolism have revealed that the metabolic reprogramming of immune cells, their resident tissues, and even the entire organism plays a crucial role in driving and sustaining chronic inflammatory responses [6,7,8]. In contrast to traditional strategies targeting single inflammatory mediators, interventions aimed at key metabolic pathways, such as glucose, lipid, and amino acid metabolism, have the potential to fine-tune inflammation at a multi-target and upstream level, offering new therapeutic avenues for autoimmune diseases like RA [9,10,11]. For instance, lactate, an immunometabolic signaling molecule, has been shown to regulate immune cell function through transporter- and receptor-mediated metabolic reprogramming in various inflammatory contexts [12]. Additionally, the balance between ceramide and sphingosine-1-phosphate is a key metabolic switch that governs the activation and function of immune cells, including macrophages, and plays a central role in inflammation [13]. Dysregulated cholesterol metabolism and the formation of cholesterol crystals, which activate the NLRP3 inflammasome and amplify IL-1β-driven inflammatory cascades, have profound effects on macrophage mediated inflammation and contribute to the pathogenesis of atherosclerosis and other chronic inflammatory conditions [14]. An increasing number of studies in RA have revealed that disturbances in lipid metabolic networks, particularly the glycerophospholipid metabolism, are closely linked to disease onset, transition from early to chronic disease, and systemic inflammatory burden [15,16,17]. Glycerophospholipid-derived phosphatidylinositol species have recently been shown to control both the priming and activation of the NLRP3 inflammasome by altering the acyl-chain composition and Toll/IL-1R domain containing adaptor protein stability in macrophages [18]. In parallel, signaling through the PI3K/AKT/mTOR axis has been implicated as an upstream metabolic checkpoint that modulates NLRP3 inflammasome activation and downstream IL-1β/IL-18 release in diverse inflammatory and pharmacological models [19,20].

Nanomedicine, particularly macromolecule small molecule self-assembled nanodrug delivery systems, has demonstrated promising synergistic anti-inflammatory effects. For example, one study employed human serum albumin and superoxide dismutase to self-assemble with methotrexate, leveraging passive targeting to inflamed tissues and reactive oxygen species scavenging properties. The resulting nanoparticles showed enhanced targeting to inflammatory sites and improved therapeutic efficacy in arthritic mice, while simultaneously reducing methotrexate’s systemic toxicity [21]. Similarly, curcumin-based self-assembled nanoparticles, using peptide or protein scaffolds, demonstrated superior mucosal repair and anti-inflammatory effects compared to free curcumin in murine models of colitis [22]. In neuroinflammation, nanoparticles formed by the self-assembly of whey protein derivatives with epigallocatechin gallate (EGCG) effectively penetrated the blood–brain barrier, inhibited microglial activation, and alleviated neuroinflammation in neurodegenerative disease models [23,24,25].

In our previous work, we developed licorice protein–paeoniflorin self-assembled nanoparticles (GP-PF NPs) and systematically characterized their physicochemical properties using dynamic light scattering and transmission electron microscopy [26,27]. These nanoparticles exhibited a uniform particle size distribution, regular morphology, and a typical core–shell structure. However, whether GP-PF NPs possess biological activity in the context of experimental arthritis model treatment and whether they can provide a synergistic, multi-target pharmacological mechanism compared to monotherapy remains unclear. Therefore, this study aims to investigate, from the perspective of metabolism-inflammation regulation, the effects of GP-PF NPs on metabolic homeostasis and the PI3K/AKT/mTOR-NLRP3 signaling-related gene in adjuvant-induced arthritis (AIA) mice. By integrating in vivo pharmacodynamic evaluation, UHPLC-Orbitrap-MS-based metabolomics analysis, and molecular biological validation, this study seeks to provide pharmacological evidence for the potential development of GP-PF NPs as a novel multi-target anti-arthritic agent.

## 2. Results

### 2.1. GP-PF NPs Reduce the Paw Swelling and Synovial Inflammation in AIA Mice

**Figure 1:**
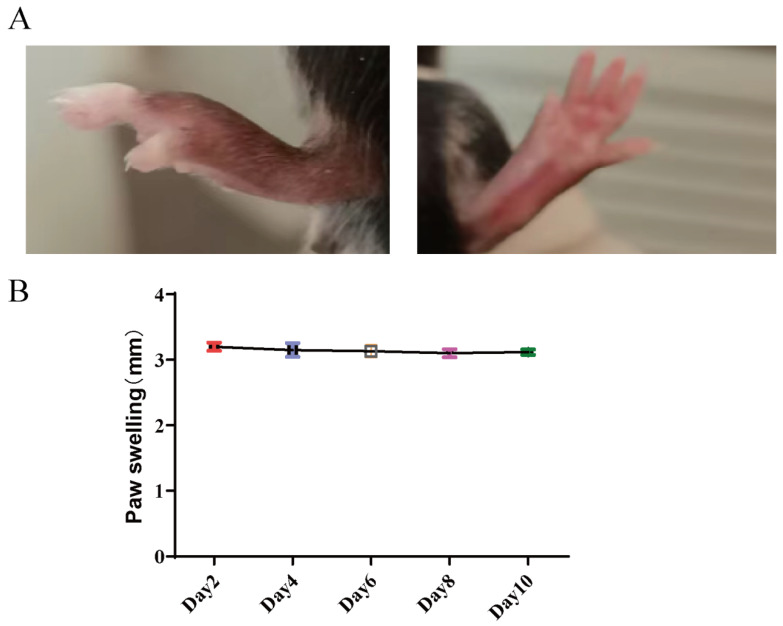
Representative paw morphology and paw swelling in the AIA model. (**A**) Representative images of the model group at Day 2 post-induction, showing severe redness and swelling of the paw with restricted movement. (**B**) Paw swelling measurement over time (Day 2, Day 4, Day 6, Day 8, and Day 10) in the model group. There is no significant change in paw swelling throughout the observation period.

In the AIA model, paw swelling was observed starting from Day 2 post-induction. Representative images of the model group at Day 2 post-induction show severe redness and swelling of the paw with restricted movement (Figure 1A). As shown in Figure 1B, the model group exhibited no significant change in paw swelling throughout the observation period, suggesting that the model group maintained a stable level of inflammation during the study period.

**Figure 2:**
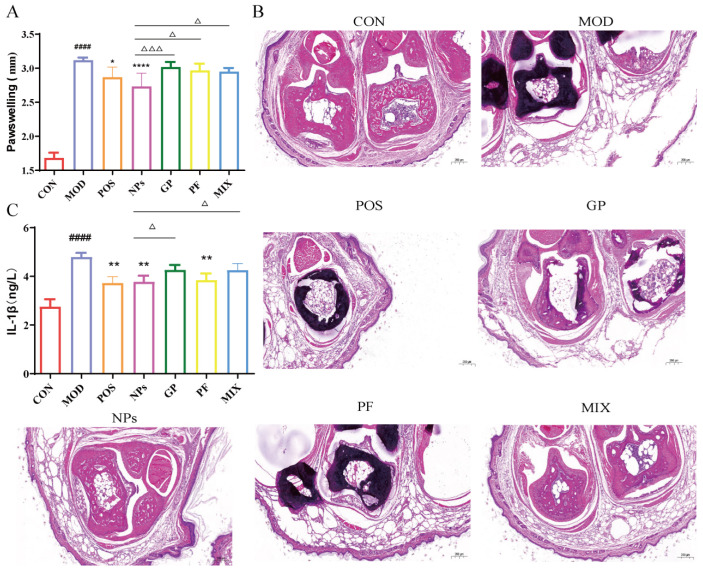
GP-PF NPs alleviate joint swelling in AIA mice and improve histopathology. (**A**) Statistical data on paw swelling. Data are presented as mean ± SD. ^####^ *p* < 0.0001 vs. control group; * *p* < 0.05, **** *p* < 0.0001 vs. model group; ^△^ *p* < 0.05, ^△△△^ *p* < 0.001 vs. GP-PF NPs group. (**B**) Representative H&E-stained sections of ankle joints (original magnification ×200). (**C**) Serum IL-1β expression level. Data are presented as mean ± SD. ^####^ *p* < 0.0001 vs. control group; ** *p* < 0.01 vs. model group; ^△^ *p* < 0.05 vs. GP-PF NPs group.

To evaluate the therapeutic efficacy of GP-PF nanoparticles, paw thickness, serum IL-1β expression, and histopathological alterations in the affected joints were assessed. As shown in Figure 2A, the paw thickness of the model group was markedly increased compared with the control group (^####^ *p* < 0.0001), indicating successful induction of arthritis. The positive control, celecoxib, alleviated paw swelling relative to the model group (* *p* < 0.05). Notably, GP-PF nanoparticles produced the strongest anti-edema effect: paw thickness in the GP-PF NPs group was lower than that in the model group (**** *p* < 0.0001) and was also reduced compared with the PF (^△^ *p* < 0.05), GP (^△△△^ *p* < 0.001), and physical mixture groups (^△△△^ *p* < 0.001), supporting an additional therapeutic benefit associated with nanoparticle formulation.

Histological assessment of ankle joints provided further corroboration of the pharmacodynamic outcomes (Figure 2B). HE staining of ankle joints in the model group revealed characteristic inflammatory lesions, marked by pronounced synovial hyperplasia, obliteration of the synovial cavity, and extensive inflammatory cell infiltration, accompanied by cartilage erosion and disorganization of the trabecular bone structure, which are indicative of typical rheumatoid like synovitis and tissue damage. Both the GP monotherapy and PF monotherapy groups exhibited moderate mitigation of pathological severity relative to the model group, including a reduction in synovial layer thickness, partial restoration of the synovial cavity patency, and diminished inflammatory cell infiltration; however, cartilage structural recovery remained limited. The physical mixture group demonstrated enhanced improvement compared to the monotherapy groups, with reduced synovial hyperplasia and inflammatory cell infiltration, along with a trend toward localized cartilage surface repair. Notably, the joint architecture in the GP-PF NPs group closely resembled that of normal tissue. The synovial layer was restored to 2–3 cell layers, the synovial cavity was patent, inflammatory cells were markedly reduced, and the cartilage surface and trabecular bone structures remained intact.

Serum IL-1β levels were shown in Figure 2C. The model group displayed elevated IL-1β compared with the control group (^####^ *p* < 0.0001), which was consistent with the robust systemic inflammation. Celecoxib lowered IL-1β, relative to the model group (** *p* < 0.01). GP-PF NPs also reduced IL-1β compared with the model group (**** *p* < 0.0001), the PF group (^△^ *p* < 0.05), and the mix group (^△^ *p* < 0.05), and further decreased it relative to the PF group (^△^ *p* < 0.05) and the mix group (^△^ *p* < 0.05).

### 2.2. GP-PF NPs Induce Global Serum Metabolic Reprogramming

To investigate the systemic metabolic changes induced by GP-PF NPs treatment, we conducted a non-targeted metabolomics analysis on the serum samples of mice. Metabolites were putatively annotated at MSI Level 2–3, based on accurate mass (mass error < 5 ppm) and MS/MS spectral similarity against mzCloud (match score ≥ 70), with retention time plausibility used as supporting evidence. Lipids for which positional isomers could not be resolved were reported as putative annotations and interpreted accordingly. In total, 473 metabolites were identified in both positive and negative ionization modes. The complete list of annotated metabolites was provided in the Supplementary Table. In addition, the mass spectrometry identification diagrams of the key metabolites was provided in Supplementary Figure S1.

**Figure 3:**
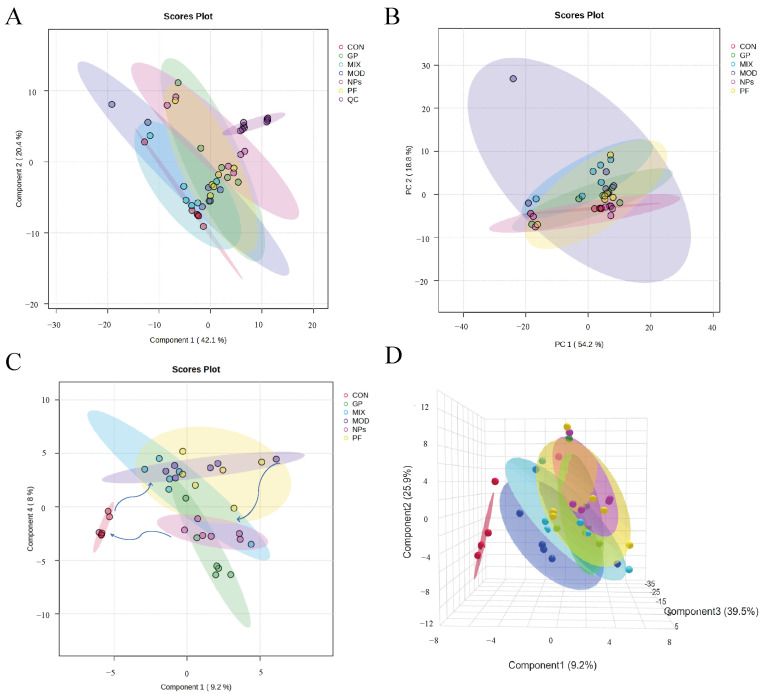
Multivariate analysis of serum metabolomic profiles in AIA and treatment groups. (**A**) PCA score plot including QC samples; (**B**) PCA score plot excluding QC samples; (**C**) 2D PLS-DA score plot of all groups; and (**D**) 3D PLS-DA score plot of all groups.

Multivariate statistical analysis was conducted to evaluate the overall metabolic differences among the various groups. As shown in Figure 3A, the PCA score plot, including QC samples, demonstrated that QC injections were closely clustered together, which suggested that the instrument has good stability and the analytical results are repeatable. After removing the QC samples, the PCA score plot (Figure 3B) showed clear separation between the control group and the other groups, indicating that arthritis induced pronounced metabolic alterations. Further discrimination was examined using supervised PLS-DA (Figure 3C,D). The 2D and 3D PLS-DA score plots showed that the control group was distinctly separated from the model group. Among the treatment groups, the separation effect of the GP-PF NPs group was the most significant compared to the model group. There was no overlap between the clusters within the model group (Figure 3C). Moreover, the NPs group showed a clear tendency to shift toward the control group, whereas other treatment groups (PF, GP, and MIX) displayed only partial divergence from the model. These findings suggest that GP-PF NPs induced the most effective metabolic restoration among all tested interventions.

**Figure 4:**
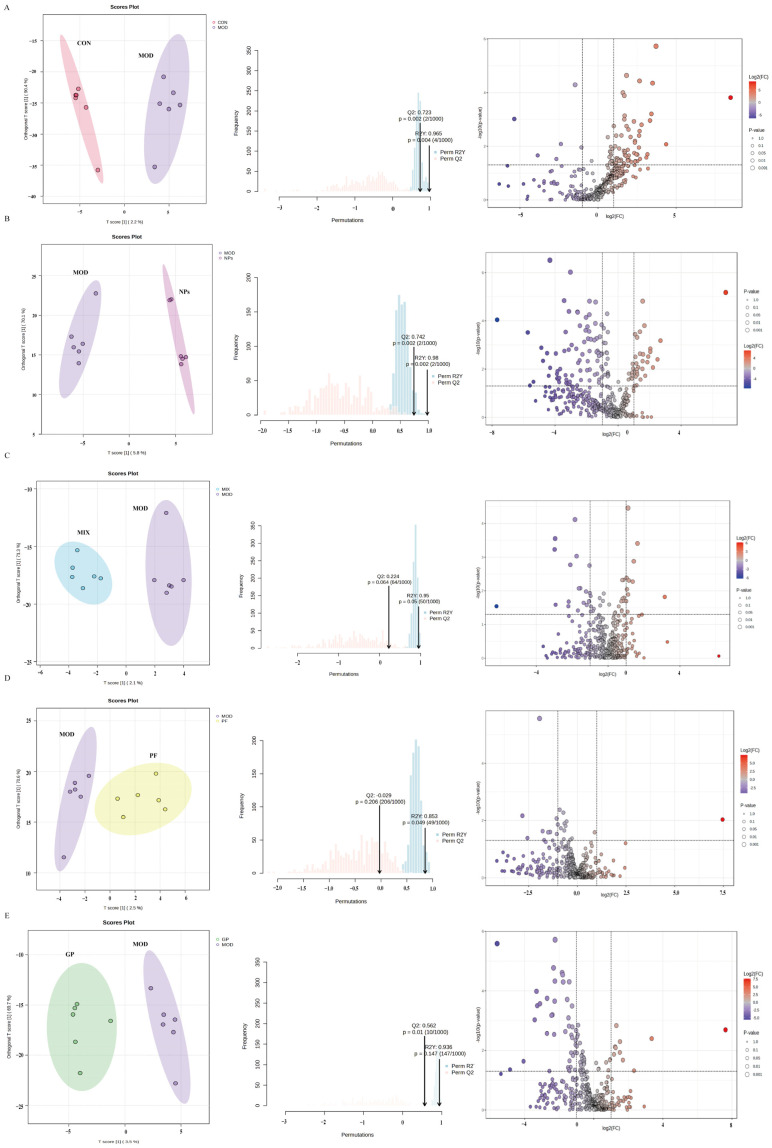
OPLS-DA models with 1000-time permutation tests and volcano plot analysis of differential metabolites between the model group and control or treatment groups. (**A**) Control vs. model: OPLS-DA score plot and permutation test (1000 permutations) supporting model validity (Q^2^ = 0.723, R^2^Y = 0.965; empirical *p* = 0.002 for Q^2^ and *p* = 0.004 for R^2^Y), together with the corresponding volcano plot; (**B**) model vs. GP-PF NPs: OPLS-DA and permutation test indicating robust discrimination (Q^2^ = 0.742, R^2^Y = 0.980; empirical *p* = 0.002 for both Q^2^ and R^2^Y) and the corresponding volcano plot; (**C**) model vs. physical mixture: moderate discrimination was observed (Q^2^ = 0.224, R^2^Y = 0.950; empirical *p* = 0.004 for Q^2^ and *p* = 0.050 for R^2^Y) and the corresponding volcano plot; (**D**) model vs. PF: limited predictability was observed (Q^2^ = 0.029, R^2^Y = 0.853; empirical *p* = 0.206 for Q^2^ and *p* = 0.049 for R^2^Y) and the corresponding volcano plot; (**E**) model vs. GP: discrimination was supported primarily by Q^2^ under permutation testing (Q^2^ = 0.562, R^2^Y = 0.936; empirical *p* = 0.010 for Q^2^ and *p* = 0.147 for R^2^Y) and the corresponding volcano plot. Volcano plots highlight significantly altered metabolites, defined as fold change > 2 with FDR-adjusted *p* < 0.05.

Using the supervised OPLS-DA model, we further distinguished the metabolic differences between the model group and each of the other groups (Figure 4A–E). The score plots showed a clear separation between the control and AIA model groups. Similarly, the GP-PF NPs group was distinctly separated from the model group, with no overlap between clusters, whereas the PF, GP, and MIX groups exhibited only partial separation.

Permutation testing with 1000 permutations showed that the observed OPLS-DA models were unlikely to be due to random class labeling for key comparisons (control vs. model: Q^2^ = 0.723, *p* = 0.002; R^2^Y = 0.965, *p* = 0.004; model vs. GP-PF NPs: Q^2^ = 0.742, *p* = 0.002; R^2^Y = 0.980, *p* = 0.002), supporting acceptable model validity under permutation testing. In contrast, comparisons between the model group and PF, GP, or the physical mixture generally exhibited weaker discrimination and reduced predictability: model vs. mix (R^2^Y = 0.950, Q^2^ = 0.224; *p* = 0.050 for R^2^Y and *p* = 0.004 for Q^2^), model vs. PF (R^2^Y = 0.853, Q^2^ = 0.029; *p* = 0.049 for R^2^Y and *p* = 0.206 for Q^2^), and model vs. GP (R^2^Y = 0.936, Q^2^ = 0.562; *p* = 0.147 for R^2^Y and *p* = 0.010 for Q^2^). These results indicate that the supervised separation was robust for control vs. model and model vs. GP-PF NPs, whereas the discriminatory performance was limited, particularly for model vs. PF, under the current sample size and validation scheme. OPLS-DA with permutation testing suggested that GP-PF NPs produced the most pronounced and statistically supported shift in the serum metabolome, relative to the model group (highest Q^2^ with significant permutation *p*-values), which was consistent with a stronger overall metabolic modulation. Nevertheless, given the modest group sizes, OPLS-DA is interpreted primarily as an exploratory visualization tool, and differential metabolites were defined using univariate statistics with FDR correction.

The results of the volcano plot analysis revealed that there were different ranges of metabolite regulation in different intervention measures. Compared with the control group, the AIA model showed 59 significant changes. The GP-PF NPs altered 108 metabolites, which was the most extensive modification among all groups, while GP and the physical mixture, respectively, regulated 38 and PF only regulated 6 metabolites (Supplementary Table).

### 2.3. GP-PF NPs Elicit a Nanoparticle-Specific Metabolic Signature Enriched in Glycerophospholipid Pathways

**Figure 5:**
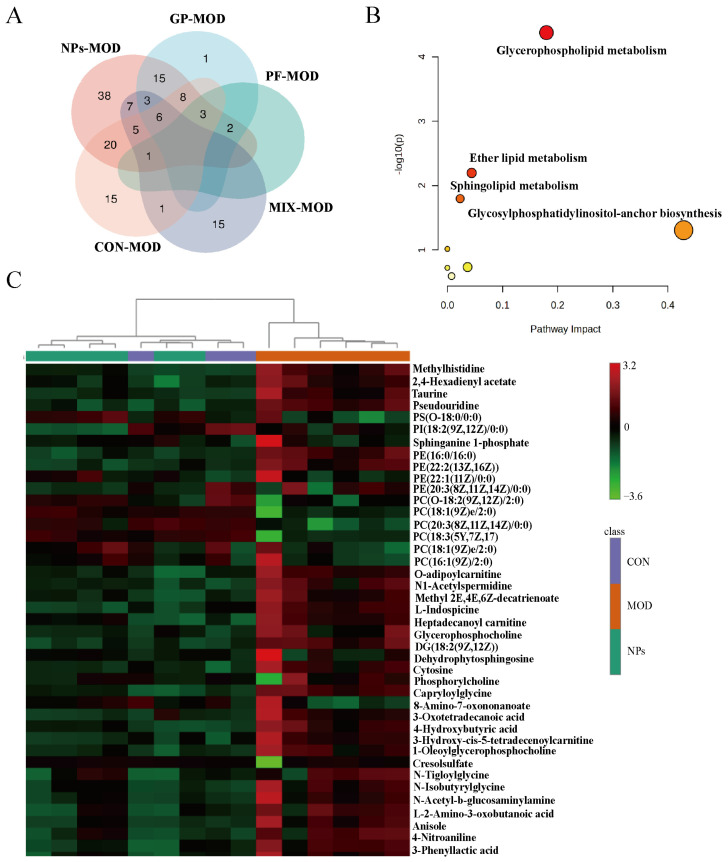
GP-PF NPs specific metabolic characteristics and pathway enrichment analysis. (**A**) The Venn diagram results showed the number of differential metabolites between each group and the model group. GP-PF NPs modulated 43 unique metabolites. (**B**) Pathway enrichment bubble plot results of 43 NP-specific metabolites. Glycerophospholipid metabolism, sphingolipid metabolism, and ether lipid metabolism were the metabolic pathways that were most significantly affected. (**C**) Hierarchical clustering heatmap of 43 GP-PF NPs-regulated metabolites across all groups.

To clarify the metabolic characteristics that are specifically regulated by GP-PF NPs, we used the Venn diagram analysis to compare the differential metabolites among different treatment groups. As shown in Figure 5A, compared with the AIA model group, a total of 43 metabolites in the NP treatment group presented unique regulatory patterns, whereas only 13, 11, and 1 metabolite were specifically altered by the physical mixture, GP, and PF treatments, respectively. These results underscore the distinctive metabolic remodeling capacity of the nanoparticle formulation. Pathway enrichment analysis was performed on the 43 NP-specific differential metabolites to explore their functional relevance. As illustrated in Figure 5B, glycerophospholipid metabolism exhibited the most significant enrichment feature, followed by ether lipid metabolism and sphingolipid metabolism. Enrichment *p*-values were FDR adjusted (top pathway FDR = 0.003367). This adjustment controls for the false discovery rate in multiple comparisons, ensuring that the observed results are less likely to be due to random chance. An FDR of 0.003367 indicates strong statistical evidence for pathway enrichment, suggesting that these pathways are robustly linked to the differential metabolites observed in the nanoparticle treatment group. The pathway impact was 0.17965 (Table 1), which reflects the relative importance of the pathway within the metabolic network; higher impact values indicate a greater contribution of the corresponding pathway to the network topology and overall metabolic perturbation. In this case, the impact value suggests that the glycerophospholipid metabolism has a notable influence on the metabolic network, which could be a key process influenced by nanoparticle treatment. Hierarchical clustering analysis (Figure 5C) further supported the unique metabolic characteristics induced by nanoparticle treatment. The clustering results indicated that the metabolic profiles of the nanoparticle-treated samples were highly similar to those of the control group, suggesting that the treatment may have subtle, yet consistent, effects on the metabolic landscape.

### 2.4. GP-PF NPs Normalize 14 Key Lipid Metabolites

**Figure 6:**
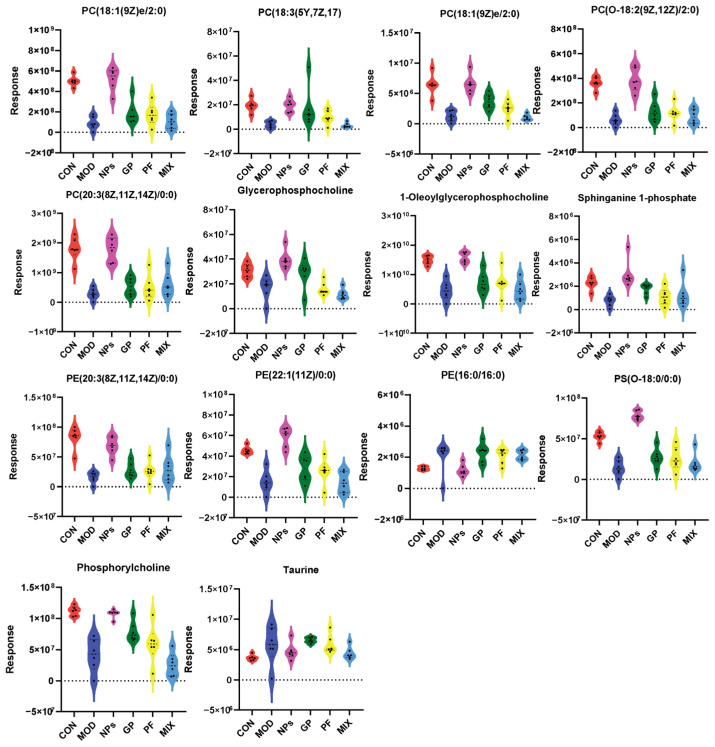
Representative violin plots of lipid markers specifically regulated by GP-PF NPs. The y-axis represents the raw mass spectrometry response intensity of the metabolites.

To deeply elucidate the metabolic mechanisms involved in the therapeutic effects of GP-PF NPs, we conducted a detailed analysis of 43 NP-specific differential metabolites. Through a violin plot analysis of all treatment groups, it was found that 14 metabolites showed significant dysregulation in the AIA model group, and these dysregulations could be selectively reversed after GP-PF NPs treatment (Figure 6).

Among these metabolites, four key compounds were involved in glycerophospholipid metabolism: glycerophosphocholine, 1-oleoylglycerophosphocholine, PE(16:0/16:0), and phosphocholine were markedly reduced in the model group but were restored toward control levels after GP-PF NPs treatment. In addition, sphingosine-1-phosphate (a key sphingolipid intermediate) and PC(18:1(9Z)e/2:0) (an ether-linked phosphatidylcholine) also shifted toward normal levels in response to GP-PF NPs. Notably, these regulatory effects were not reproduced by monotherapy with PF or GP, nor by their physical mixture.

### 2.5. GP-PF NPs Downregulate Synovial mRNA Expression of PI3K/AKT/mTOR-NLRP3 Inflammasome-Related Genes

**Figure 7:**
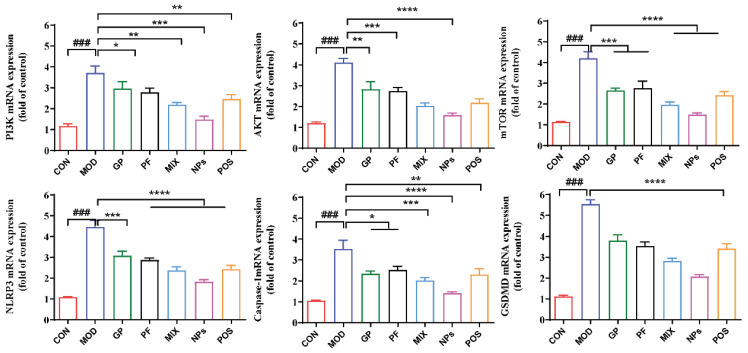
qPCR results showing mRNA expression of PI3K, AKT, mTOR, NLRP3, Caspase-1, and GSDMD in synovial tissue. Data are expressed as mean ± SD. ^###^ *p* < 0.001 vs. control group; * *p* < 0.05, ** *p* < 0.01, *** *p* < 0.001, and **** p < 0.0001 vs. model group.

To investigate the anti-inflammatory mechanism of GP-PF NPs at the molecular level, qPCR was performed to assess the mRNA expression levels of key components involved in the PI3K/AKT-NLRP3 signaling pathway within the synovial tissue. As shown in Figure 6, the expression levels of six key genes: PI3K, AKT, mTOR, NLRP3, Caspase-1, and GSDMD were significantly upregulated in the AIA model group compared to the control group (^###^ *p* < 0.001), suggesting a marked transcriptional upregulation of PI3K/AKT/mTOR axis components and inflammasome-related genes in the model group. Compared with the model group, GP-PF NPs markedly downregulated the transcription of PI3K/AKT/mTOR axis genes, with PI3K showing the most pronounced reduction toward basal levels. In parallel, GP-PF NPs also substantially suppressed NLRP3 inflammasome-related genes, including NLRP3, Caspase-1, and GSDMD, indicating an overall inhibition of the PI3K/AKT/mTOR-NLRP3 inflammatory cascade at the mRNA level. Notably, the physical mixture (MIX) and the single components (GP or PF) generally exhibited weaker or partial reductions compared with GP-PF NPs, while the positive control group also showed significant decreases in pathway-associated transcripts (Figure 7).

### 2.6. Serum Metabolites Associated with Synovial Gene Expression

**Figure 8:**
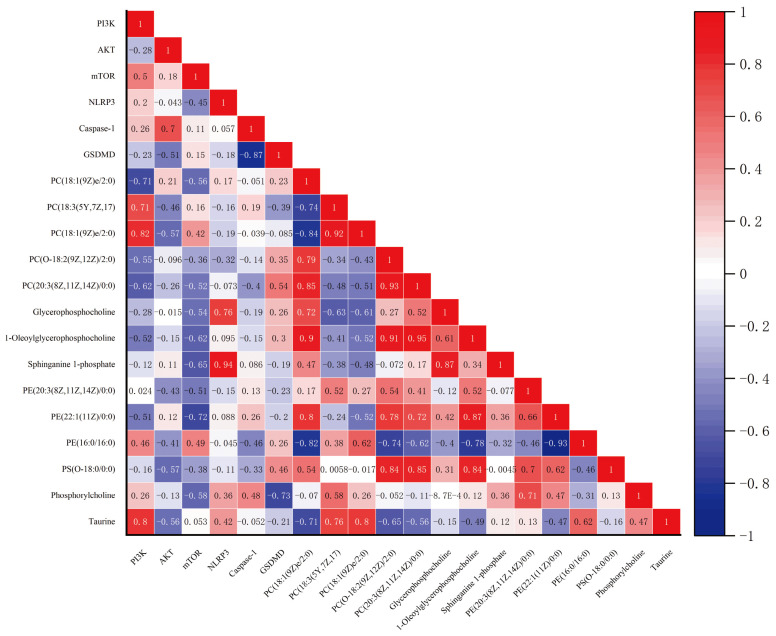
Associations between Selected Serum Metabolites and Synovial Gene Expression Levels.

To further investigate the relationship between the 14 serum metabolites regulated by the GP-PF NPs and 6 synovial gene expressions, we performed correlation analysis. As shown in Figure 8, notably, PI3K demonstrated strong positive correlations with metabolites such as PC(18:3(5Y,7Z,17) and PC(18:1(9Z)e/2:0), Taurine, with correlation coefficients of 0.71 and 0.71, 0.82,0.8, respectively, and AKT was negatively correlated with these metabolites, with correlation coefficients of 0.71 and −0.46, −0.57,−0.56, respectively. This suggests that the activation of PI3K/AKT signaling may be associated with higher levels of certain phospholipids in the serum. mTOR showed a weaker but still significant correlation with sphinganine 1-phosphate and glycerophosphocholine (r = −0.65 and r = −0.54, respectively), indicating potential interaction between metabolic regulation and mTOR pathway signaling. NLPR3 was positively correlated with several lipid metabolites, including sphinganine 1-phosphate (r = 0.91) and glycerophosphocholine (r = 0.76), suggesting that inflammation markers may be associated with alterations in lipid metabolism. Interestingly, Caspase-1 and GSDMD, key genes involved in inflammasome activation and pyroptosis, showed negative correlations with multiple phospholipids. The correlation matrix further revealed significant relationships among various lipid metabolites, such as sphinganine 1-phosphate and glycerophosphocholine (r = 0.87), sphingaine 1-phosphate and PC(18:1(9Z)/2:0) (r = 0.94), and PC(20:3(8Z,11Z,14Z)0:0) with PS(O-18:0/0:0) (r = 0.85), highlighting strong metabolic interconnectivity between lipid molecules in the systemic environment.

## 3. Discussion

To our knowledge, this is the first study to evaluate GP-PF NPs in AIA by integrating metabolomic readouts with inflammatory signaling markers. Our results suggest that the potential therapeutic mechanism of GP-PF NPs in AIA is linked to the glycerophospholipid metabolism and PI3K/AKT/mTOR-NLRP3 inflammatory signaling, evidenced by glycerophospholipid remodeling and reduced synovial pathway gene transcription.

In the paw swelling and serum IL-1β assessment, the GP-PF NPs group showed the greatest anti-inflammatory effect, relative to free PF, GP monomer, and their physical mixture. Consistent with this finding, a histopathological examination of joint tissues indicated that the GP-PF NPs group exhibited the mildest synovial hyperplasia and inflammatory cell infiltration. Notably, the GP monomer also produced measurable improvements in paw swelling, IL-1β levels, and synovitis, supporting its intrinsic pharmacological activity as a functional biomacromolecule, and acts not only as a nanocarrier but also as an active component involved in inflammatory regulation [26]. Although the physical mixture showed better efficacy than either single component alone, its overall benefit remained limited, indicating that simple co-administration may be insufficient to achieve maximal therapeutic enhancement. Together, these results suggest that the rational nanostructural design and molecular level assembly of GP and PF may contribute to improved anti-arthritic efficacy beyond physical mixing.

To further investigate the synergistic therapeutic mechanism of GP-PF NPs, we compared the overall regulatory capacity of different interventions on serum metabolites in AIA mice. Nontargeted metabolomics analysis showed that the GP-PF NPs group could significantly reverse 43 differential metabolites in the AIA model, whereas the free GP and PF groups and the physical mixture group reversed only 11, 1 and 13 metabolites, respectively. This result, at the systems level, supports that the nanoassembly strategy confers GP-PF NPs with a broader metabolic regulatory capacity, rather than a mere superimposed effect of each component.

Through the box plot analysis, we found that among the 43 key metabolites specifically regulated by GP-PF NPs, 14 metabolites exhibited a stronger reversal toward the control phenotype than the comparator groups (single agent and physical mixture groups), where these metabolites displayed only limited modulation, forming a candidate GP-PF NPs-specific metabolic signature. Notably, lipid metabolites dominated among these 14 substances, including 1-oleoylglycerophosphocholine, glycerophosphocholine, sphingosine-1-phosphate, and others. These metabolites are implicated in inflammatory and lipid signaling processes that are relevant to AIA pathophysiology. Plasma glycerophosphocholine and related lysophospholipids are decreased in RA and inversely correlate with disease-activity-related inflammatory markers (such as erythrocyte sedimentation rate, C-reactive protein, and IL-6), indicating an association between glycerophospholipid metabolic disturbance and inflammation in RA [15,16,28,29]. Sphingosine-1-phosphate, as an important bioactive sphingolipid, binds to its receptor’s S1PR1–5 to regulate lymphocyte egress, macrophage polarization, and angiogenesis, and has been implicated in synovitis and joint destruction in RA [30,31,32,33]. Moreover, RA-related metabolomics studies have reported that decreased 1-oleoyl-sn-glycero-3-phosphocholine (OGPC), a lysophospholipid, is associated with higher plasma IL-6. In vitro, OGPC supplementation inhibits TNF-α induced IL-6 secretion and JAK2 phosphorylation in synovial cells, suggesting that restoring OGPC may have anti-inflammatory potential and may help to attenuate pro-inflammatory signaling, such as the JAK/STAT pathway [15,16]. Our data provide additional evidence for glycerophospholipid-related metabolic abnormalities in the inflammatory stage of AIA.

Interestingly, although the physical mixture showed a degree of anti-inflammatory activity (such as reduced paw swelling, IL-1β, and joint inflammation, its ability to modulate glycerophospholipid-related metabolic alterations was limited and did not reproduce the extent of regulation of metabolites, such as 1-oleoylglycerophosphocholine and glycerophosphocholine, observed with GP-PF NPs. This phenomenon indicates that ordered assembly at the nanoscale confers unique advantages for achieving multi-target metabolic regulation, especially the glycerophospholipid metabolites. Previous studies have suggested that precisely engineered nanostructures can enhance synergistic regulation of complex metabolic networks by optimizing the spatiotemporal distribution and release kinetics of active components [34,35,36,37]. Taken together, our findings further support that constructing nanoparticles by self-assembling GP and PF not only enhances the overall pharmacological efficacy, but also promotes broader correction of disordered glycerophospholipid metabolism, exceeding the effects of single components or simple co-administration.

Glycerophospholipid metabolic reprogramming has been reported to be closely linked to the aberrant activation of key signaling pathways such as PI3K/AKT/mTOR. In pathological contexts, including cancer and chronic inflammation, bidirectional crosstalk and feedback interactions between these processes have been described [38,39]. Among glycerophospholipid-derived lipid mediators, phosphoinositides, particularly phosphatidylinositol (PI) derivatives, serve as core second messengers in the PI3K/AKT cascade by generating PI(3,4,5)P_3_ and recruiting the PH domain containing PDK1 and Akt, and then directly drive activation of this pathway [40,41]. In addition, the sphingolipid mediator sphingosine-1-phosphate plays important roles in inflammatory processes in rheumatic diseases by binding to its G protein-coupled receptors (S1PR1–5), thereby regulating lymphocyte trafficking, vascular permeability, and downstream inflammation-related signaling networks [30,42]. Based on this evidence, we hypothesized that GP-PF NPs associated glycerophospholipid metabolic remodeling is linked to the therapeutic response, and it occurs in parallel with reduced synovial transcription of genes in the PI3K/AKT/mTOR-NLRP3 axis. To test this hypothesis, we quantified synovial mRNA expression of PI3K/AKT/mTOR pathway genes. GP-PF NPs decreased PI3K, AKT, and mTOR transcripts compared with the AIA model group. Consistently, GP-PF NPs also reduced the NLRP3, Caspase-1, and GSDMD mRNA levels in synovial tissue compared with model group, indicating suppressed transcriptional activity of PI3K/AKT/mTOR and inflammasome-related signaling. This transcriptional pattern was consistent with our metabolomics findings and suggests an association between glycerophospholipid remodeling and PI3K/AKT/mTOR-related signaling, which was further supported by the correlation analysis results.

By integrating pharmacodynamic, metabolomic, and molecular evidence, our study demonstrates that GP-PF NPs confer greater anti-arthritic benefit in the AIA model than PF alone or a PF-GP physical mixture. Mechanistically, this advantage is accompanied by a nanoassembly-specific glycerophospholipid remodeling signature and reduced synovial mRNA expression of PI3K/AKT/mTOR-NLRP3 axis components. This multilevel, multi-target mechanistic analysis provides preclinical support for developing metabolism-inflammation oriented multi-target therapeutic strategies in experimental arthritis. Meanwhile, the specific glycerophospholipid metabolites identified in this study (such as glycerophosphocholine and 1-oleoylglycerophosphocholine) may be informative for monitoring metabolic and inflammatory states in experimental arthritis.

This study has several limitations. First, we used a single animal model (CFA-induced AIA) in male C57BL/6 mice with a relatively small sample size. Although this model is widely used to study inflammatory arthritis, the generalizability of our findings to other RA models, to female animals, and to larger cohorts or more chronic disease settings remains to be established. In particular, potential sex-specific differences in treatment responses warrant further investigation. Second, the treatment duration and endpoint assessment were relatively short. Longer-term studies are required to evaluate the durability of efficacy, structural protection, and safety of GP-PF NPs, including their effects on joint degeneration, immune regulation, and tissue repair. Third, dose–response relationships were not systematically assessed. Although equivalent doses were used across treatment groups to isolate formulation-related effects, nanoparticle delivery may modify the dose–response profile and contribute to the apparent efficacy advantage; this should be addressed in future studies using multi-dose designs. Fourth, the untargeted metabolomics observations should be followed by targeted, quantitative assays and independent validation of key lipid metabolites to establish their reproducibility and potential as biomarkers. Finally, mechanistic validation in this study was primarily performed at the transcript level, using RT-qPCR. Additional confirmation at the protein level (e.g., Western blotting and immunohistochemistry) and functional assays (e.g., inflammasome activation, IL-18 release, ASC speck formation, and cleavage of caspase-1 and GSDMD) will be important to strengthen causal interpretation. Future work will incorporate these protein level and functional readouts to provide more definitive evidence linking PI3K/AKT signaling and glycerophospholipid metabolic remodeling to the observed therapeutic effects.

## 4. Materials and Methods

### 4.1. Materials and Chemicals

Paeoniae Radix Alba (*Paeonia lactiflora* Pall.) and Glycyrrhizae Radix et Rhizoma (*Glycyrrhiza uralensis* Fisch.) were purchased from Beijing Tongrentang Pharmacy (Beijing, China). The crude drugs were authenticated as genuine materials in accordance with the standards of the Pharmacopoeia of China (2020 Edition) by Prof. Chunsheng Liu (Beijing University of Chinese Medicine). Standard reference substances of paeoniflorin (purity ≥ 98%) were obtained from the National Institutes for Food and Drug Control (Beijing, China). Complete Freund’s adjuvant (CFA) and celecoxib were purchased from Sigma-Aldrich (St. Louis, MO, USA). TRIzol™ reagent and the PrimeScript™ RT Reagent Kit were obtained from Takara Bio Inc. (Kusatsu, Japan). All other reagents and solvents were of analytical or LC-MS grade and used as received without further purification.

### 4.2. Preparation of GP-PF Nanoparticles

GP-PF nanoparticles were prepared via a heat-induced self-assembly strategy. Briefly, GP (the preparation method of GP can be found in the previous literature [27]) and PF were mixed at a mass ratio of 10:1 and dissolved in phosphate-buffered saline (PBS, pH 7.4). The solution was stirred at 200 rpm and titrated with 1% NaOH until the pH reached 8.0. The mixture was then heated at 100 °C for 40 min under continuous stirring (300 rpm) to facilitate nanoparticle formation. After cooling to room temperature, the solution was dialyzed against ultrapure water, using a membrane with a molecular weight cut-off (MWCO) of 8–14 kDa for 12 h, with water replacement every 3 h. The dialyzed solution was frozen at −80 °C and lyophilized to obtain GP-PF nanoparticle powder. To ensure reproducibility, the nanoparticles were synthesized in three independent batches and subjected to quality control analysis. The average size, PDI, zeta charge, and in vivo stability data of GP-PF nanoparticles were shown in Supplementary Figures S2–S4. Additionally, the preparation method for the physical mix group was the same as that of the nano group, which is at a mass ratio of 10:1 mixture, but without heating.

### 4.3. Animal Model and Experimental Design

A total of 42 male C57BL/6 mice (6–8 weeks old, 18–22 g, SPF grade at purchase) were obtained from SiPeiFu Biotechnology Co. (Beijing, China). The sample size was determined based on prior experience and feasibility; no a priori calculation was performed. Only male C57BL/6 mice were used, to minimize the variability associated with the estrous cycle and sex-hormone fluctuations, which may affect inflammatory and metabolic readouts in experimental arthritis. Animals were housed under controlled conditions (temperature 22 ± 2 °C, humidity 50 ± 10%, 12 h light/dark cycle) in a clean-grade animal facility, with free access to standard chow and water. All animal procedures were performed in accordance with the ethical standards approved by the Institutional Animal Ethics Committee of the Institute of Basic Theory, China Academy of Chinese Medical Sciences (approval number: IBTCMCACMS21-2403-18, date: 20 March 2024).

Mice were randomly assigned into seven groups, using a random number table method to ensure unbiased group allocation. The groups were as follows: (1) control group (*n* = 6), receiving normal saline; (2) AIA model group (*n* = 6), also receiving normal saline; (3) GP-PF NPs group (*n* = 6), treated with glycyrrhiza protein–paeoniflorin nanoparticles; (4) physical mixture group (*n* = 6), receiving a combination of paeoniflorin and glycyrrhiza protein in solution (*n* = 6); (5) paeoniflorin group (*n* = 6), treated with paeoniflorin alone; (6) glycyrrhiza protein group, treated with glycyrrhiza protein alone; and (7) positive control group (*n* = 6), administered celecoxib. AIA was induced in all groups except the control by subcutaneous injection of 50 μL CFA into the left hind paw. From Day 2 post-induction, mice received daily intraperitoneal injections of their respective treatments for 10 consecutive days. Drug doses were calculated based on equivalent content: 0.6 mg/10 g body weight for paeoniflorin, 6 mg/10 g for glycyrrhiza protein, and 0.3 mg/10 g for celecoxib. The control and model groups received equal volumes of sterile normal saline. Confounders were minimized by standardizing housing and procedures; rotating cage position; performing dosing measurements at a fixed time of day; and randomizing the daily order of handling across groups. Analgesia was administered according to the institutional animal welfare protocol, except where contraindicated. Humane endpoints were predefined: mice were euthanized under anesthesia if they showed >15–20% body weight loss or inability to access food or water. No animals met the humane endpoint criteria and no unexpected adverse events occurred.

Inflammation was monitored starting from Day 2 post-induction. The clinical inflammation scoring was based on a 0–4 scale. The model group monitored the foot swelling data every two days. On Day 11, paw thickness was measured using a precision digital caliper (accuracy: 0.01 mm) to monitor the progression of inflammation. Mice were euthanized under anesthesia, and blood and joint tissues were harvested for subsequent histological, metabolomic, and molecular analyses. To reduce potential confounding, AIA induction, drug administration, and paw thickness measurement were performed at a consistent time of day, using a standardized protocol. Animals were to be excluded a priori if they died before the planned endpoint, showed severe intercurrent illness unrelated to AIA induction, or if sample collection failed (e.g., insufficient volume or gross hemolysis). No animals were excluded from the analyses.

### 4.4. Pharmacodynamic and Histological Assessment

Therapeutic efficacy was evaluated based on paw swelling and histopathological changes in ankle joints. Ankle joints were dissected and fixed in 4% paraformaldehyde for 24 h at 4 °C, followed by decalcification in 10% EDTA solution for 14 days at 4 °C. After routine dehydration and paraffin embedding, joints were sectioned at 4 μm thickness and stained with hematoxylin and eosin (H&E) for histological evaluation.

Histological assessments focused on synovial hyperplasia, inflammatory infiltration, and cartilage destruction. Sections were examined under an Olympus BX53 light microscope (Tokyo, Japan) by two independent observers who were blinded to group allocation. Paw thickness measurement and data analysis were performed by investigators who were blinded to group allocation, using the coded sample.

### 4.5. Determination of Serum IL-1β Expression

Serum levels of IL-1β were quantified using a commercially available enzyme-linked immunosorbent assay (ELISA) kit, which was specific for mouse IL-1β (Jiangsu Meimian Industrial Co., Ltd., Yancheng, China); catalog no. MM-0040M1), according to the manufacturer’s instructions. Standards and serum samples were added in duplicate to wells pre-coated with anti-mouse IIL-1β capture antibody. After incubation at room temperature for 2 h, wells were washed three times with wash buffer, and a biotinylated detection antibody was added and incubated for 1 h. Following additional wash steps, streptavidin-horseradish peroxidase (HRP) conjugate was applied for 30 min. After a final series of washes, tetramethylbenzidine (TMB) substrate was added, and the enzymatic reaction was stopped with sulfuric acid. Optical density was measured at 450 nm, using a microplate reader. A standard curve was generated from serial dilutions of recombinant mouse IL-1β, and sample concentrations were calculated by fitting absorbance values to the curve. All measurements fell within the linear range of the standard curve, and intra- and inter- assay coefficients of the variation were kept below 10%.

### 4.6. Serum Metabolomics Analysis

#### 4.6.1. Sample Collection and Preparation

Following the final treatment on Day 10, mice were fasted for 12 h and lightly anesthetized. Blood was collected via orbital enucleation, clotted at room temperature for 1 h, and centrifuged at 3000 rpm for 10 min at 4 °C. Serum was separated, aliquoted, and stored at −80 °C until analysis.

For metabolomics, 50 μL thawed serum was mixed with 200 μL pre-chilled (−20 °C). LC-MS grade acetonitrile was used to precipitate proteins, vortexed for 30 s, incubated at 4 °C for 30 min, and centrifuged at 13,000 rpm for 10 min at 4 °C. The supernatant was evaporated to dryness under vacuum and reconstituted in 50 μL 50:50 (*v*/*v*) acetonitrile/water, vortexed, and recentrifuged prior to injection.

A pooled quality-control (QC) sample was prepared by combining 2 μL from each study sample and was injected after every six study injections to monitor instrument stability and analytical reproducibility.

#### 4.6.2. UHPLC-Orbitrap-MS Conditions

Serum profiling was performed on a Vanquish™ UHPLC coupled with an O Exactive™ Orbitrap mass spectrometer (Thermo Scientific, Waltham, MA, USA). Separation used a Hypersil GOLD C18 column (2.1 mm × 100 mm, 1.9 μm) at 40 °C. Mobile phases were 0.1% formic acid in water (A) and 0.1% formic acid in acetonitrile (B); gradient: 0–5 min, 2–25% B; 5–18 min, 25–70% B; 18–27 min, 70–98% B; 27–28 min, 98% B; and 29–30 min, return to 2% B. The flow rate was 0.3 mL/min and the injection volume was 5 μL.

Data were acquired in both positive and negative ESI. Full MS with ddMS2 was used over *m*/*z* 100–1200. Typical settings: spray voltage 3200 V (pos)/2500 V (neg); ion-transfer-tube 325 °C; capillary 350 °C; and sheath/auxiliary gas 50/10 (arb). Full MS resolution 60,000 FWHM and MS/MS resolution 30,000 FWHM with stepped NCE 20/40/60 eV. Raw files were acquired in Xcalibur and converted to mzXML for processing.

Prior to scaling, total ion chromatogram (TIC)-based normalization was applied. QC performance was evaluated by the median RSD of non-blank QC features (target < 30%) and by tight QC clustering in unsupervised PCA score plots.

#### 4.6.3. Data Processing and Analysis

Raw data were processed in Compound Discoverer 3.1 (Thermo Scientific) for peak detection, alignment, retention time correction, and noise filtering. Feature filtering removed isotopes/fragments/adducts where applicable. Prior to statistical analysis, features with excessive missingness (e.g., present in < 80% of samples within at least one group) were removed, and the remaining missing values were imputed using a small value approach (half of the minimum positive value per feature) after normalization.

Metabolite annotation used mzCloud, mzVault, and ChemSpider. Separate libraries were built for positive and negative modes and then combined. For each reported differential metabolite, we provide the library/source, entry/ID, precursor/product ions, mass error (<5 ppm), and MS/MS match score (mzCloud match ≥ 70).

The data matrix was log transformed and Pareto-scaled. PCA (unsupervised) was used to inspect the overall clustering, QC tightness, and batch effects; sample level quality was assessed using QC performance (median RSD of non-blank QC features < 30% and tight QC clustering in PCA), and no biological samples were excluded from the metabolomics analysis. OPLS-DA (supervised) was performed for pairwise comparisons (model vs. each treatment). Model performance was reported as R^2^Y and Q^2^, with CV-ANOVA *p*-values and 1000-time permutation tests to assess overfitting; volcano plots were used to visualize discriminating variables. Differential metabolites were defined based on a combination of statistical significance (FDR-adjusted *p* < 0.05) and fold-change thresholds (log2 fold change > 2) by volcano plot analysis. Pathway analysis was performed on the MetaboAnalyst website and enrichment *p*-values were adjusted by BH-FDR; detailed parameters can be found in Supplementary Figure S5. To ensure blinding, all serum samples were assigned anonymized alphanumeric codes by an independent investigator. Sample preparation and LC-MS injection order were randomized across groups, and data processing and statistical analyses were performed on coded datasets. Group identities were revealed only after the primary analyses were completed.

### 4.7. qPCR Analysis of PI3K/AKT/mTOR-NLRP3-Related Gene

The total RNA was extracted from the synovial tissue of the left ankle joint, using TRIzol™ reagent (Kusatsu, Japan) in accordance with the manufacturer’s instructions. RNA concentration and purity were assessed using a NanoDrop 2000 spectrophotometer (Thermo Fisher Scientific, USA), and samples with an A260/A280 ratio between 1.8 and 2.0 were selected for further use.

Complementary DNA (cDNA) was synthesized from 1 μg of RNA using the PrimeScript™ RT Reagent Kit (Takara, Japan). Quantitative real-time PCR (qPCR) was performed using a QuantStudio 5 Real-Time PCR System (Applied Biosystems, Waltham, MA, USA) and SYBR Green Master Mix at a total volume of 10 μL, including 0.2 μM of each primer. The thermal cycling program consisted of an initial denaturation at 95 °C for 30 s, followed by 40 cycles of denaturation at 95 °C for 5 s and annealing/extension at 60 °C for 30 s.

Primers targeting PI3K, AKT, mTOR, NLRP3, Caspase-1, and GSDMD were designed and synthesized by Shanghai Sangon Biological Engineering Co., Ltd. (Shanghai, China). The housekeeping gene GAPDH was used as the internal reference. Primer sequences are provided in Table 2. All reactions were run in technical triplicate. A melt curve analysis was performed following amplification to verify the specificity of each product.

Relative mRNA expression levels were calculated using the 2^−ΔΔCt^ method. Expression values were normalized to the control group and reported as fold change. To ensure blinding, tissue samples were labeled with anonymized codes. Samples were randomly assigned to plate positions, and result calculations were performed using coded sample IDs. Group allocation was unblinded only after completion of the analyses.

### 4.8. Statistical Analysis

All quantitative data are presented as mean ± standard deviation (SD). Statistical analysis was performed using GraphPad Prism 9.0 (GraphPad Software Inc., San Diego, CA, USA). Data normality was assessed by the Shapiro–Wilk test, and the homogeneity of variances was checked using Levene’s test (or the Brown–Forsythe test for non-normal data). If assumptions were violated, one-way ANOVA with Tukey’s post hoc test was applied. If the normality and homogeneity of variances were not satisfied, Kruskal–Wallis test with Dunn’s post hoc test was used. A *p*-value < 0.05 was considered statistically significant unless otherwise specified. Unless otherwise stated, *n* = 6 mice per group were used for paw thickness measurement, histology, serum metabolomics, and qPCR. qPCR reactions were run in technical triplicate, which were not treated as independent biological replicates.

### 4.9. Correlation Analysis of Serum Metabolites and Synovial Gene Expression

To investigate potential associations between systemic metabolic changes and local inflammatory gene expression, correlation analyses were performed between 14 quantified serum metabolites and the expression levels of six synovial genes. Prior to analysis, all variables were tested for normality using the Shapiro–Wilk test in Origin 2022 (OriginLab Corporation, Northampton, MA, USA). Data that deviated markedly from normal distribution were log-transformed to satisfy parametric test assumptions. Pearson’s correlation coefficients (r) were calculated for normally distributed datasets, while Spearman’s rank correlation was applied when one or more variables did not meet the normality criteria.

## 5. Conclusions

By integrating in vivo pharmacodynamic evaluation with untargeted metabolomics and molecular evidence, this study demonstrates that self-assembled GP-PF nanoparticles confer superior anti arthritic efficacy in the CFA-induced AIA model compared with PF monotherapy or the physical mixture of PF and GP, as reflected by improvements in paw swelling, joint histopathology, and serum 1β levels. Metabolomics further indicates a nanoassembly specific lipid remodeling signature induced by GP-PF NPs, predominantly involving glycerophospholipid- and sphingolipid-related metabolites, accompanied by reduced synovial transcription of genes within the PI3K/AKT/mTOR-NLRP3 axis, supporting an association between lipid remodeling and inflammatory signaling regulation. Overall, ordered nanoscale assembly may enable coordinated metabolic-inflammatory modulation beyond simple co-administration; however, multi-dose and longer term studies, targeted quantification, and protein level validation are still required to further substantiate the proposed mechanism and biomarker utility.

## Figures and Tables

**Table 1:** Pathway enrichment results.

Pathway Name	Match Status	*p*	FDR	Impact
Glycerophospholipid metabolism	4/36	0.000042084	0.0033667	0.17965
Ether lipid metabolism	2/20	0.0063549	0.25419	0.04403
Sphingolipid metabolism	2/32	0.015933	0.42489	0.02313
Taurine and hypotaurine metabolism	1/8	0.049266	0.98533	0.42857
Histidine metabolism	1/16	0.096337	1	0
Glycosylphosphatidylinositol-anchor biosynthesis	1/32	0.18424	1	0.03665
Glycine, serine and threonine metabolism	1/33	0.18947	1	0
Primary bile acid biosynthesis	1/46	0.25476	1	0.00758

**Table 2:** Primer sequence targeting PI3K, AKT, mTOR, NLRP3, Caspase-1, and GSDMD.

Gene (Mouse)	Forward Primers (5′-3′)	Reverse Primers (5′-3′)
PI3K	ACCACGAGTCTCTCGCTCAGTA	CCTGATACTGAGAGTGGAACTCC
AKT1	GGACTACTTGCACTCCGAGAAG	CATAGTGGCACCGTCCTTGATC
mTOR	AGAAGGGTCTCCAAGGACGACT	GCAGGACACAAAGGCAGCATTG
NLRP3	TCACAACTCGCCCAAGGAGGAA	AAGAGACCACGGCAGAAGCTAG
Caspase1	GGCACATTTCCAGGACTGACTG	GCAAGACGTGTACGAGTGGTTG
GSDMD	GGTGCTTGACTCTGGAGAACTG	GCTGCTTTGACAGCACCGTTGT
GAPDH	CATCACTGCCACCCAGAAGACTG	ATGCCAGTGAGCTTCCCGTTCAG

## Data Availability

All data generated or analyzed in this study are available from the corresponding author upon reasonable request.

## Supplementary Materials

The following supporting information can be downloaded at: https://www.mdpi.com/article/10.3390/molecules31030554/s1.

## Abbreviations

PF	Paeoniflorin
GP	Glycyrrhiza protein
GP-PF NPs	Glycyrrhiza protein–paeoniflorin nanoparticles
RA	Rheumatoid arthritis
TNF-α	Tumor necrosis factor-alpha
IL-1β	Interleukin-1β
AIA	Adjuvant-induced arthritis
CFA	Complete Freund’s adjuvant
LC-MS	Liquid chromatography-mass spectrometry
PBS	Phosphate-buffered saline
PI3K	Phosphatidylinositol 3-Kinase
AKT	protein kinase B (Akt/PKB)
mTOR	Mechanistic target of rapamycin
NLRP3	NOD-like receptor pyrin domain-containing 3
Caspase-1	Cysteine-aspartic protease-1
GSDMD	Gasdermin D
ANOVA	Analysis of variance
H&E	Hematoxylin and eosin (staining)
