# Supplementary material for: A UHPLC-Orbitrap-MS Metabolomics Strategy Reveals Glycerophospholipid Metabolic Remodeling Is Associated with the Anti-Arthritic Effect of Glycyrrhiza Protein–Paeoniflorin Nanoparticles via PI3K/AKT/NLRP3 Axis
Source: Molecules. 2026 Feb 5;31(3):554. doi: 10.3390/molecules31030554 (PMC12899346; doi:10.3390/molecules31030554)
Supplement: Supplementary file 1 [file molecules-31-00554-s001.zip › Supplementary Figures.pdf]

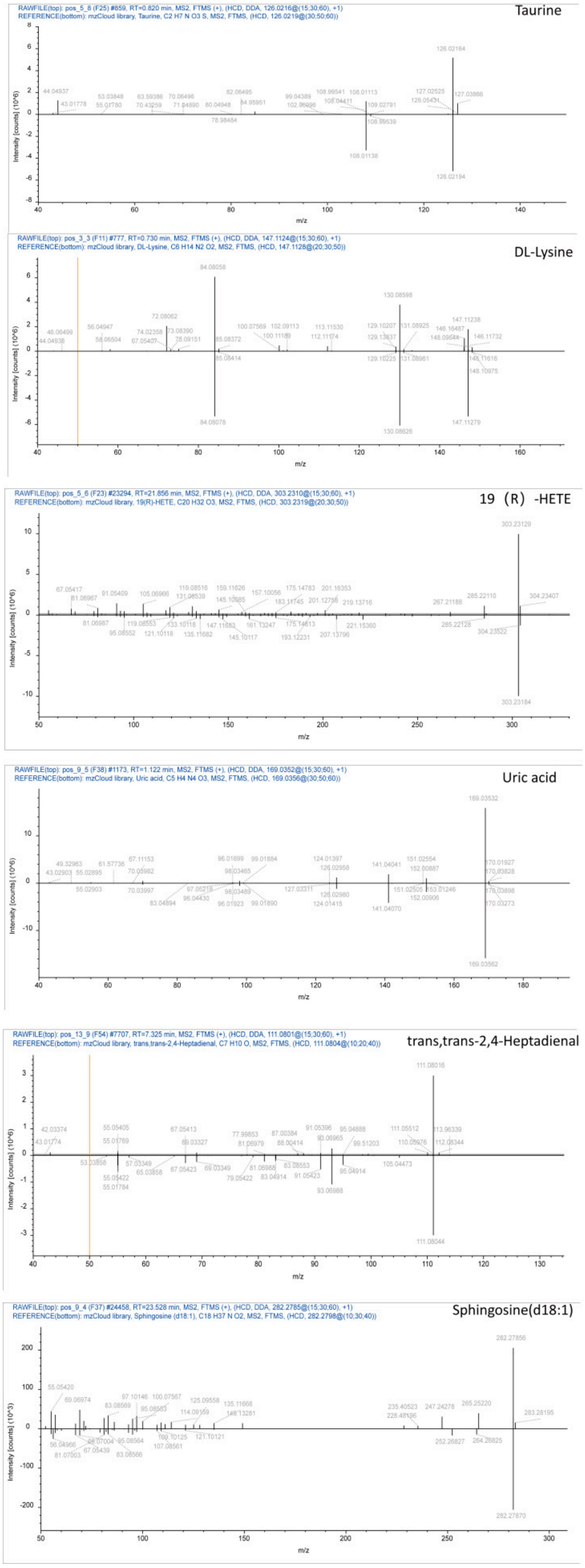

Supplementary Figure S1. Identification results of representative metabolites' mass spectra and their matching with the mzcloud database image

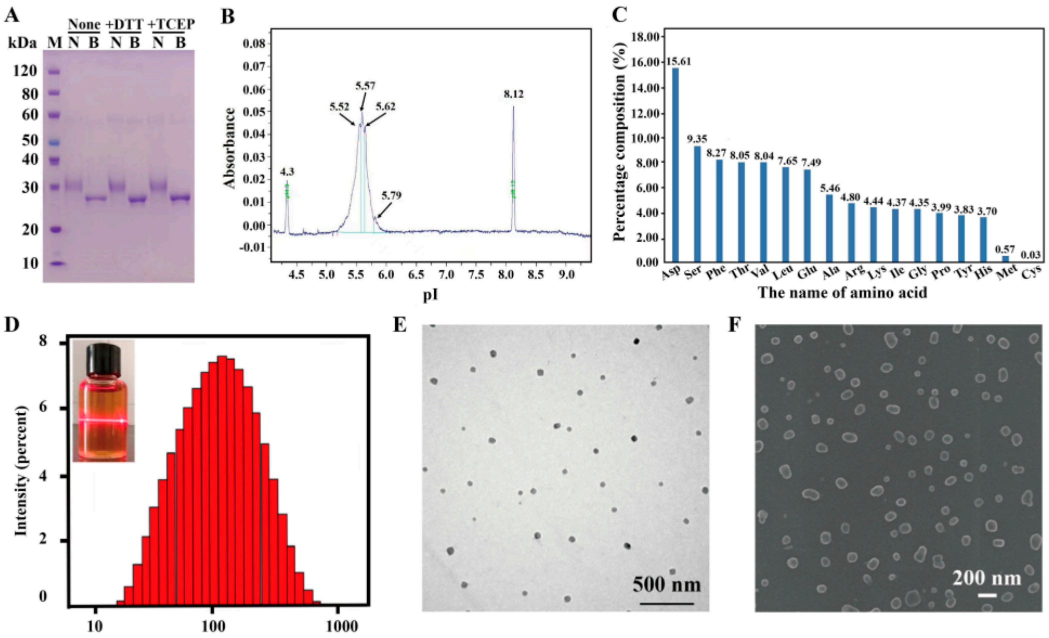

Supplementary Figure S2. Characterization of GP and GP-PF NPs. (A) SDS-PAGE analysis of purified GP under different treatment conditions: Lane 1, native GP; Lane 2, heat-treated GP; Lane 3, GP with 0.1M DTT; Lane 4, heat-treated GP with 0.1 M DTT; Lane 5, GP treated with 0.1 M TCEP; and Lane 6, heat-treated GP with 0.1 M TCEP. (B) Isoelectric point diagram of GP. (C) Amino acid composition of GP. (D) Tyndall effect of GP-PF NPs in aqueous solution and particle size map of GP. (E) Transmission electron microscopy (TEM) images of GP-PF NPs. (F) Scanning electron microscopy (SEM) images of GP-PF NPs. Abbreviations: GP, Glycyrrhiza protein; GP-PF NPs, Glycyrrhiza protein–paeoniflorin nanoparticles; SDS-PAGE, Sodium dodecyl sulfate polyacrylamide gel electrophoresis; DTT, Dithiothreitol; TCEP, Tris(2-carboxyethyl)phosphine.

The Particle Size and Zeta Potential of GP, PF, the Mixture of GP and PF, and GP-PF NPs

	Particle Size (nm)	PDI	Zeta Potential (mv)
PF	1.02±0.0082	0.068 ± 0.011	0.62±0.0000
GP	19.96±1.7242	0.127 ± 0.031	1.74±0.0365
Mixture	21.31±0.3001	0.421 ± 0.029	1.71±0.0200
GP-PF NPs	148.23±0.0436	0.269 ± 0.027	-(84.98±3.0795)

Notes: (n = 4, mean ± standard deviation).
Abbreviations: PF, Paeoniflorin; GP, *Glycyrrhiza* protein; GP-PF NPs, *Glycyrrhiza* protein–paeoniflorin nanoparticles; PDI, polydispersity index.

Supplementary Figure S3. Characterization of GP and GP-PF NPs.

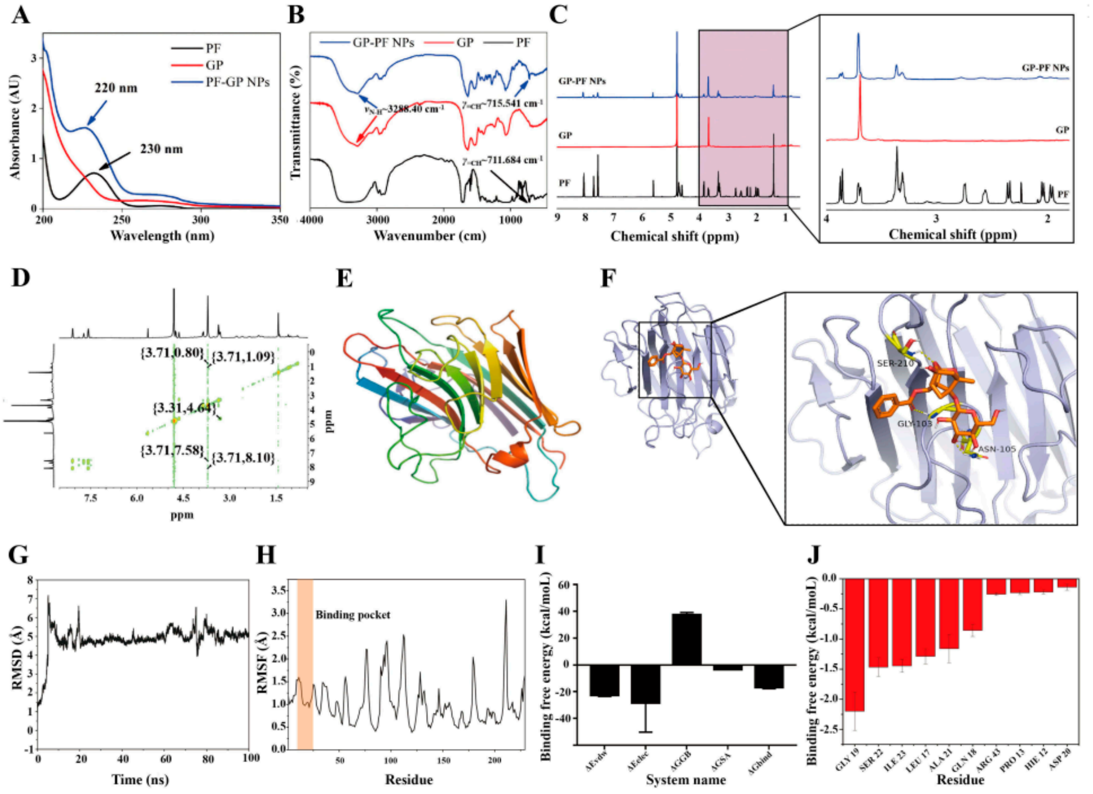

Supplementary Figure S4. Characterization of GP and GP-PF NPs. Self-assembly and molecular interactions of GP-PF NPs. (A)UV－Vis spectra of GP, PF, and GP-PF NPs. (B) FT-IR spectra of GP, PF, and GP-PF NPs. (C) 1H-NMR spectra of GP, PF, and GP-PF NPs. (D) COSY spectra of GP-PF NPs. (E) Homology-modeled GP structure used for molecular docking studies. (F) Binding interactions of PF within GP’ s binding pocket, highlighting key hydrogen bonds and hydrophobic interactions. (G) RMSD curves of GP and PF in 100 ns molecular dynamics simulation. (H) RMSF analysis diagram of the GP during the 200 ns molecular dynamics simulation. (I) The binding free energy of the GP-PF NPs system. (J) Contribution of the top 10 amino acid residues to GP-PF NPs binding affinity.

Specify pathway analysis parameters:

Visualization method	◉ Scatter plot (testing significant features)<br>○ Heatmaps (testing your selected features)
Enrichment method	◉ Hypergeometric Test<br>○ Fisher's Exact Test
Topology measure	◉ Relative-betweeness Centrality<br>○ Out-degree Centrality
Reference metabolome	◉ Use all compounds in the selected pathway library<br>○ Upload your own reference metabolome

Enrichment method	[illegible]<br>○ Fisher's Exact Test
Topology measure	◉ Relative-betweeness Centrality<br>○ Out-degree Centrality
Reference metabolome	◉ Use all compounds in the selected pathway library<br>○ Upload your own reference metabolome

ect a pathway library (KEGG pathway info were obtained in Dec. 2024). Scroll to find more options in each panel.

Mammals [20]	◉ Homo sapiens (KEGG)<br>○ Homo sapiens (SMPDB)<br>○ Pan troglodytes (chimpanzee) (KEGG)<br>○ Macaca mulatta (rhesus monkey) (KEGG)
Birds [2]	○ Gallus gallus (chicken) (KEGG)<br>○ Taeniopygia guttata (zebra finch) (KEGG)
Fish [2]	○ Danio rerio (zebrafish) (KEGG)<br>○ Nothobranchius furzeri (turquoise killifish) (KEGG)

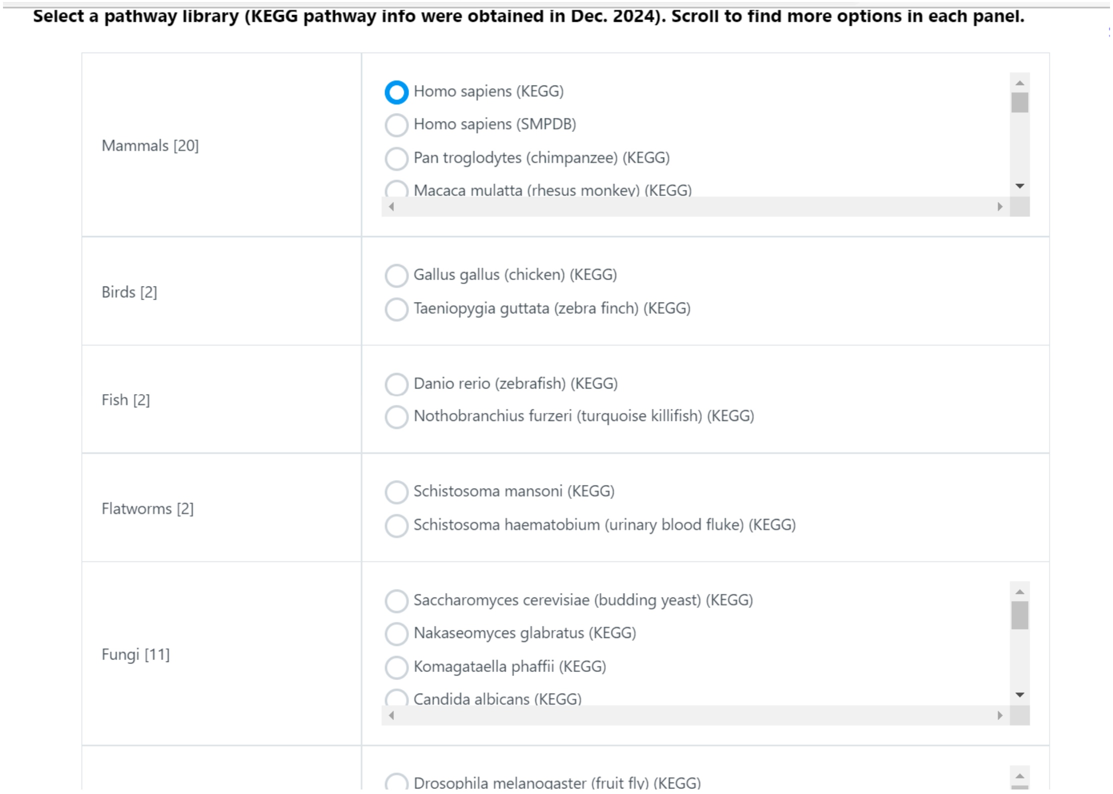

Supplementary Figure S5. Pathway enrichment analysis parameters of the MetaboAnalyst software
